# The transcriptome of CDK4/6 inhibition

**DOI:** 10.18632/aging.101285

**Published:** 2017-08-30

**Authors:** Erik S. Knudsen, Agnieszka K. Witkiewicz

**Affiliations:** Department of Medicine, University of Arizona, Tucson, AZ 85721, USA

**Keywords:** cyclin D1, RB, E2F, retinoblastoma, palbociclib, abemaciclib, ribociclib, CDK4/6

***The transcriptome of CDK4/6 inhibition implication for therapeutic response, senescence, and uncharted biological complexit.*** CDK4/6 inhibitors have been recently FDA approved for the treatment of breast cancer and are incorporated into a plethora of ongoing clinical trials [1]. While there is considerable knowledge regarding the cell cycle inhibitory action of such agents, a global understanding of the response to pharmaceutical CDK4/6 inhibition is only starting to emerge. In recently published studies the use of gene expression profiling is beginning to elucidate the overall effects of these agents on tumor cells that represent both canonical, as well as unexpected responses that have significance for therapeutic intervention [2-4].

**Figure 1:**
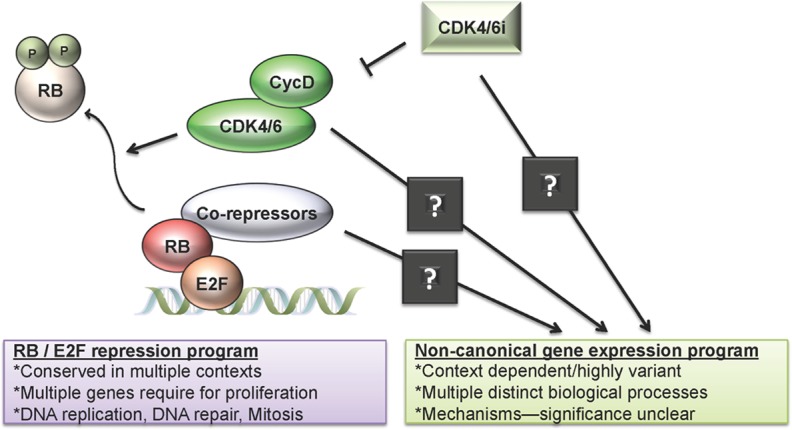
Transcriptional responses to CDK4/6 inhibition The pharmaceutical inhibition of CDK4/6 results in two distinct series of transcriptional programs. First, CDK4/6 inhibition results in the blockade of RB phosphorylation and enhanced RB-mediated transcriptional repression. This gene expression program is highly conserved and involves multiple genes that are “universally” required for proliferation. These genes play critical roles in DNA replication, DNA repair, and mitotic progression. In contrast, there is poorly understood cadre of genes that are regulated as a consequence of CDK4/6 inhibition. The mechanisms and pathways that lead to the induction of these genes are poorly understood, as is their significance to the functional effects of CDK4/6 inhibition. Specifically, they could represent off-target effects from the inhibitors, additional non-canonical targets for CDK4/6, or context specific targets for the RB tumor suppressor.

***CDK4/6 inhibitors elicit potent suppression of genes required for cell cycle progression.*** That CDK4/6 inhibition results in suppression of cell cycle regulated genes is not particularly surprising [3]. This response is dependent on the presence of the RB-tumor suppressor and reinforces the concept that the text-book pathway from CDK4/6, through RB, to E2F transcriptional repression is consistent with CDK4/6 inhibitor treatment (FIG 1). However, what is emerging is that this response is particularly constant across different disease models and different classes of CDK4/6 inhibitors. Notably, the agents palbociclib and abemaciclib were developed on different chemical scaffolds, exhibit distinct kinase inhibitory activity, and harbor distinct toxicity profiles and dosing schedules in the clinic. In spite of these differences, both agents serve to largely suppress the same genes both in cell culture and in xenograft models [3]. Such data serves as the basis for generating overall response signatures that could serve as quantitative pharmacodynamics markers for drug efficacy in a tissue agnostic setting.

Specific analyses of the genes repressed by CDK4/6 inhibition reveal important points regarding the function of CDK4/6 inhibitors and means to further develop novel combination approaches [2, 3]. First, the signature of cell cycle regulated genes is often associated with poor prognosis in specific diseases (e.g. estrogen receptor positive breast cancer). Thus, it can be envisioned that in such contexts the CDK4/6 inhibitor will serve to “normalize” gene expression and perhaps convert the disease toward a more indolent and treatable form [2]. Second, CDK4/6 inhibition results in the suppression of a host of genes that are essential for cellular viability and proliferation. For example, CDK4/6 inhibition results in the suppression of PLK1, CDC20, CCNB1, CCNA2, CDC45 and other genes that are fundamentally required for DNA replication or mitosis [3]. Ostensibly, as long as these genes are fully suppressed a CDK4/6 inhibitor will block proliferation. These data also suggest that combining a CDK4/6 inhibitor with agents that would serve to reinforce transcriptional repression will be particularly fruitful. This could represent an explanation for the robust clinical activity with endocrine therapy in ER-positive breast cancer. Alternatively, the diminished level of select genes could represent a novel pharmaceutically induced vulnerability. For example, repression of thymidylate synthase could represent a sensitizing event for 5-fluorouracil sensitivity. Further, preclinical study will be required to robustly support these mechanisms towards clinical application.

***Non-canonical targets of CDK4/6 inhibition and intrinsic complexity of the transcriptional response***. While CDK4 and CDK6 kinase activity is currently viewed as largely regulating cell cycle dependent process via phosphorylation of RB, unbiased analysis reveals a host of genes that are modulated by CDK4/6 inhibitors that seemingly have no direct connection to cell cycle (Figure 1). In particular, genes that are up-regulated as a consequence of CDK4/6 inhibition cannot be clearly ascribed to any feature of cell cycle control [2-4]. Additionally, unlike the common nature of suppressed genes, upregulated genes are context dependent and differ significantly even between breast cancer cell lines. The mechanisms driving these gene expression changes and the overall significance remains unclear. Since chronic CDK4/6 inhibition in some instances can induce features of senescence it is possible that some of the elements being observed are consistent with a senescence associated secretory phenotype (SASP). However, while the induction of classical SASP genes and a strong senescent phenotype has been observed in melanoma models [5], in breast cancer there is only a weak senescence response and the genes that are induced are not within the SASP signature. Perhaps most importantly, studies with neoadjuvant exposure to CDK4/6 inhibitors suggest that even with ∼16 weeks of exposure, withdrawal of drug prior to surgery enables cell cycle re-entry [6]. Thus, at least in the context of ER-positive breast cancer there is little functional evidence for “clinical senescence”.

The nature of upregulated genes and whether they contribute to cell cycle arrest, resistance to CDK4/6 inhibition, or some other features of biology remain under study. It has been shown that RB can contribute to a variety of non-cell cycle responses, including features of immune response and metabolic reprogramming [4, 7, 8]. Similarly, adaptation to CDK4/6 inhibition can impinge on multiple signaling pathways that can have relevance to the durability of response, and can direct novel combination approaches [4, 7]. The diversity of these responses and context-dependence clearly adds to the complexity of the biological state induced by CDK4/6 inhibition. However, understanding the mechanisms/pathways will need to be addressed to fully capitalize on therapeutic potential of CDK4/6 inhibition in the clinic.
